# Incidence of Stevens-Johnson Syndrome and Toxic Epidermal Necrolysis: A Nationwide Population-Based Study Using National Health Insurance Database in Korea

**DOI:** 10.1371/journal.pone.0165933

**Published:** 2016-11-11

**Authors:** Min-Suk Yang, Jin Yong Lee, Jayeun Kim, Gun-Woo Kim, Byung-Keun Kim, Ju-Young Kim, Heung-Woo Park, Sang-Heon Cho, Kyung-Up Min, Hye-Ryun Kang

**Affiliations:** 1 Institute of Allergy and Clinical Immunology, Seoul National University Medical Research Center, Seoul, Korea; 2 Department of Internal Medicine, Boramae Medical Center, Seoul National University College of Medicine, Seoul, Korea; 3 Department of Internal Medicine, Seoul National University College of Medicine, Seoul, Korea; 4 Public Health Medical Service, Boramae Medical Center, Seoul National University College of Medicine, Seoul, Korea; 5 Institute of Health and Environment, Seoul National University, Seoul, Korea; 6 Drug Safety Monitoring Center, Seoul National University Hospital, Seoul, Korea; San Gallicano Dermatologic Institute, ITALY

## Abstract

**Background:**

Stevens-Johnson syndrome (SJS) and toxic epidermal necrolysis (TEN) are life-threatening diseases; however, it is hard to estimate their incidence due to the rarity of these diseases. We evaluated the incidence of SJS and TEN using a nationwide administrative database.

**Methods:**

We used a national medical insurance review system (Health Insurance Review and Assessment) database which contained the claim data of the entire nation from 2009 to 2013 to estimate the accurate incidence of SJS and TEN in Korea. The diagnostic codes of L511 (SJS) or L512 (TEN) from the International Classification of Diseases-10th revision were used to define the target study population. We also retrospectively followed up a 2011 SJS and TEN cohort for 24 months in order to assess the in-hospital mortality, related complications and total claims cost due to SJS and TEN.

**Results:**

A total of 1,167 (938 SJS and 229 TEN) cases were newly diagnosed from 2010 to 2013. The age- and sex-standardized annual incidences estimated in this study were 3.96 to 5.03 in SJS and 0.94 to 1.45 in TEN per million. There was no significant change in annual incidence throughout the study periods. When analyzed by 10-year age groups, the annual incidence was the lowest in group 20–29 years and the highest in group 70 for both SJS and TEN. Based on the 2011 cohort analysis, the in-hospital mortality were 5.7 and 15.1% for SJS and TEN, respectively. The mortality increased with age, particularly, after 40 years of age. Among the complications related with SJS or TEN, ocular sequelae was the most common (43.1 and 43.4% of SJS and TEN patients, respectively) followed by urethral sequelae (5.7 and 9.4% of SJS and TEN patients, respectively).

**Conclusion:**

Overall, our data suggest that SJS, and TEN are infrequent but constantly arise throughout the years.

## Introduction

Stevens-Johnson syndrome (SJS) and toxic epidermal necrolysis (TEN) are rare but severe cutaneous adverse reactions (SCARs) which cause significant morbidity and mortality. The annual incidence of SJS and TEN in the general population is known to be 1–6 and 0.4–1.2 per million people, respectively [1–4]. The mortality rates associated with SJS and TEN were estimated as 1–5% and 30%, respectively [5]. Although several hospital based incidences of SJS and TEN have been estimated, epidemiologic data, which could represent the incidence in the general population, has been lacking [6–8]. Moreover, because SJS and TEN are rare diseases for which a large general population is needed to estimate the incidence, hospital data based incidence assumptions have limitations in their accuracy. Difficulties in obtaining definitive diagnoses of SJS and TEN are also a hurdle in estimating an accurate incidence. The diagnoses of SJS and TEN have been controversial in the past, and erythema multiforme major (EMM) has been often confused with SJS and TEN until an expert consensus group suggested a consensus clinical classification [9]. Now, most of the cases with SJS and TEN are considered to be associated with medications in contrast to the association of EMM with infections such as the herpes simplex virus [10, 11]. International Classification of Diseases-10th Revision (ICD-10) also deleted a code for EMM and specified codes for bullous conditions such as SJS (L511) and TEN (L512) versus non-bullous erythema multiforme (L510).

Epidemiologic data can provide important information to physicians and public health professionals to help understand the basis of health problems and to establish an appropriate health care plan. However, precise epidemiologic studies are costly as well as difficult to carry out especially for extremely rare diseases such as SJS or TEN. Fortunately, Korea has a nationwide disease database that includes all the people of the entire country based on a single mandatory government-established nationwide insurance system. This database is suitable for incidence because it consists of the entire national population, i.e., denominator and existing cases of diseases and numerator in the same system.

This study was done to evaluate the updated incidence, in-hospital mortality, and related complications of SJS and TEN using the National Health Insurance database in Korea.

## Materials and Methods

### Data Sources and Study Populations

Korea has adopting a single mandatory government-established health insurance system, the National Health Insurance (NHI) which is covering about 97% of the Korean people. The other 3% are supported by a Medical Aid Service (MAS) which is run by the government for low income individuals. As of 2011, there were 50,908,646 of the total Korean population, composing of 49,299,165 (96.8%) NHI beneficiaries and 1,609,481 (3.2%) MAS recipients [12]. The Health Insurance Review and Assessment Service (HIRA) is an independent Korean government agency responsible for evaluating medical claim data from all healthcare organizations and medical facilities. HIRA has accumulated the entire medical records about Korean people. In case of 2011, HIRA gathered 1.3 billion of medical claims from the entire Korean citizens. Finally, study population of this study was the entire Korean populations (approximately 50 million people) and we used the HIRA database from 2009 to 2013, in order to determine the annual incidence of SJS and TEN [13].

### Patient Selection Process

**Fig 1:**
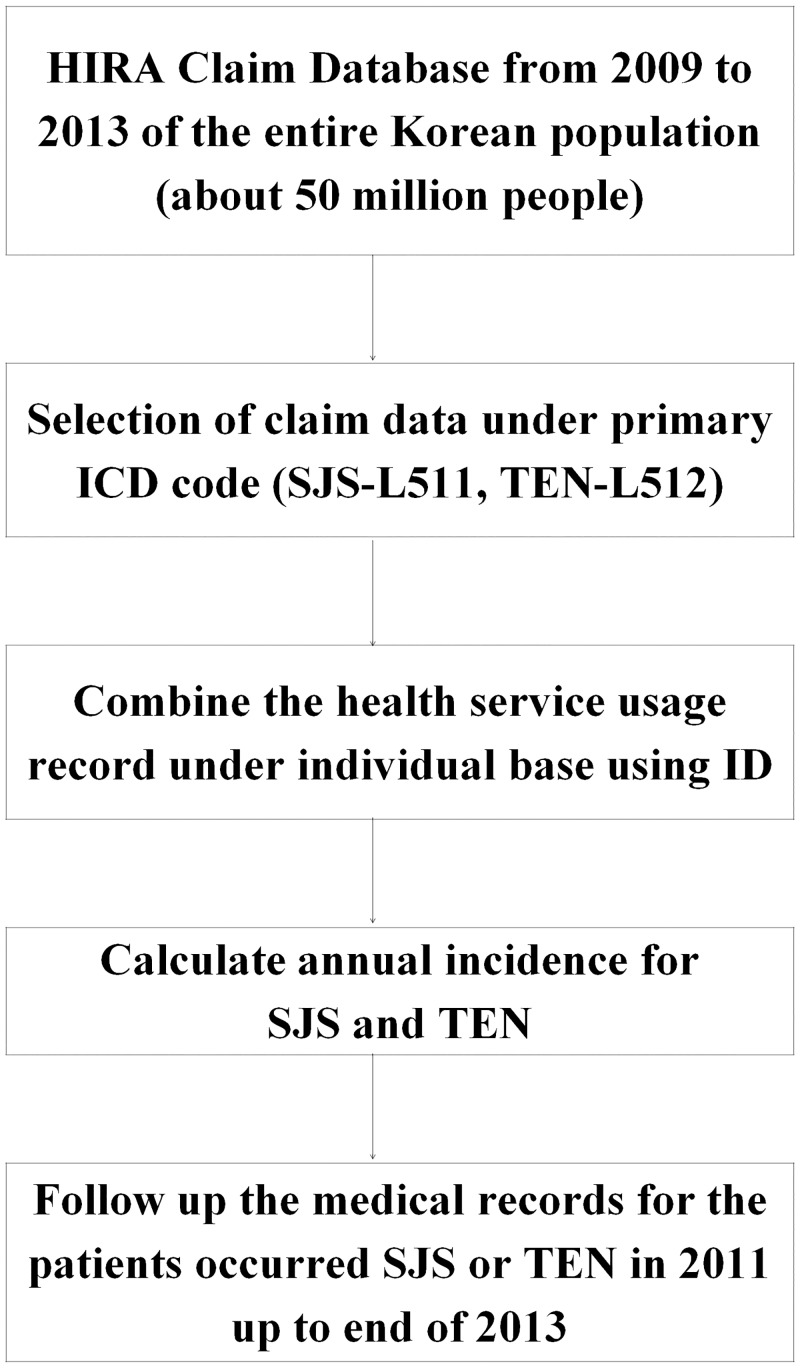
The framework of this study.

We used HIRA claim database from 2009 to 2013 in order to identify patients with SJS and patients with TEN. The patients with SJS or TEN were defined as those who had an admission history with L511 (SJS) or L512 (TEN) as a primary diagnostic code in the International Classification of Diseases, 10th revision (ICD-10). In particular, SJS or TEN patients who had a primary diagnostic code of diseases that should be differentiated from SJS and TEN such as Staphylococcal scalded skin syndrome (L00) or bullous disorders (L10-L14) later during admission or after discharge were excluded because the diagnosis of SJS or TEN at first time would be inaccurate in these cases. Fig 1 shows the framework of this study.

### Calculating Annual Incidence of SJS and TEN

Medical Subject Headings (MeSH) defines incidence as “the number of new cases of a given disease during a given period in a specific population”. In this study, “a specific population” refers the Korean population and “a given period” means each year from 2010 to 2013, respectively. In order to define “new cases”, the year of 2009 was set up the wash-out period. We gathered annual incidence cases from 2010 to 2013, respectively. For example, the incidence of SJS in 2010 means the number of inpatients with SJS who were first diagnosed in 2010 without any treatment or diagnosis history of L511 or L512 in 2009 among the entire Korean population. The incidence of SJS in 2013 refers the number of inpatients with SJS who were first diagnosed in 2013 without any treatment or diagnosis history of L511 or L512 from 2009 to 2012 among the entire Korean population. We calculated incidence in two ways such as crude incidence and standardized incidence per one million people. Crude incidence means the calculation results without any adjustment whereas standardized incidence refers the calculation results with sex and age adjustment. For standardization, we used the population data from the Korea National Statistics Office and the Health Insurance Statistical Yearbook published by the National Health Insurance Service [13]. We also used the population data from the Organization for Economic Co-operation (OECD) for standardization for international comparison [14].

### Establishing Retrospective Cohort Database Based On Incidence data of 2011

In order to investigate in-hospital mortality, possible sequelae, and medical costs, we established retrospective cohort database based on the incidence data of 2011. There were 246 patients with SJS and 53 TEN patients in the 2011 incidence data. We merged the 2011 incidence data and their medical record from 2009 to 2013. This is because we thought the possible sequelae should be observed for 24 months before and after from the onset time to confirm the presence of the sequelae and to avoid misjudgment of preexisting medical condition as sequelae. Therefore, we constructed ‘newly diagnosed SJS and TEN in 2011 cohort’ and followed up for two years in order to assess the in-hospital mortality, possible sequelae, and medical costs of 2011. In-hospital mortality was calculated as total number of mortality cases of SJS or TEN in 2011 divided by total number of admissions due to SJS or TEN in 2011. To assess the occurrence of complication or sequelae, additional diagnostic codes for the patients in the cohort during the admission periods and outpatient visits after discharge were collected. Possible complications or sequelae were defined as L81 (other disorders of pigmentation), L60 (nail disorder), H00-H59 (diseases of the eye and adnexa), N35 (urethral stricture), N36 (other disorders of the urethra), N37 (urethral disorders in diseases classified elsewhere), N39 (Other disorders of the urinary system), N46 (male infertility), N48 (other disorders of the penis), N49 (inflammatory disorders of male genital organs, not elsewhere classified), N70-N77 (inflammatory diseases of female pelvic organs), N88-N98 (non-inflammatory disorders of the female genital tract), and K12 (stomatitis and related lesions). If the patients had same diagnostic codes within 1 year before they were diagnosed with SJS or TEN, those codes were not counted as complication or sequelae. We analyzed total claim costs for SJS and TEN. Total claim costs can be divided by the costs reimbursed from the National Health Insurance Service in Korea (NHIS) and out-of-pocket (OOP) expense paid by the beneficiaries. All costs are presented in U.S. dollars (USD), with an exchange rate of 1 USD equal to 1,107.9 Korean won (average annual rate in 2011).

### Statistical Analysis

Frequency analyses were done to describe the incidence of SJS and TEN from 2010 to 2013. We conducted a Pearson's chi-square test to identify any differences between variables. Chi-square tests for trends were also performed to verify whether there were linear tendencies by years. All analyses were done with SAS Enterprise Guide (SAS Institute, Inc., Cary, North Carolina). All statistical tests were two-sided, and a p-value <0.05 was considered statistically significant.

### Ethics Statement

This study was exempted from approval by the institutional review board of Seoul National University Hospital (IRB No. 1501-088-642).

## Results

There were 938 SJS and 229 TEN cases of newly diagnosed SJS and TEN from 2010 to 2013, respectively. Age- and sex-standardized incidence rates (SIR) of SJS and TEN in 2010 were 4.88 and 0.94 per million, respectively. The annual SIR of SJS and TEN in 2011, 2012 and 2013 was 4.96, 3.96 and 5.03 and 1.08, 1.45 and 1.12 per million, respectively (Table 1). There was no time-trend change in the incidence of SJS and TEN during the 4-year study period (p for trend 0.95 for SJS and 0.85 for TEN).

**Table 1:** Incidence of Stevens-Johnson syndrome and toxic epidermal necrolysis from 2010 to 2013 in Korea.

			2010	2011	2012	2013	P for trend
SJS	No. of cases	Total	235	246	200	257	0.88
	Standardized incidence (Korea)*	Total	4.88	4.96	3.96	5.03	0.95
	Standardized incidence (OECD)†	Total	5.48	5.81	5.00	5.89	0.97
TEN	No. of cases	Total	45	53	73	58	0.09
	Standardized incidence (Korea)*	Total	0.94	1.08	1.45	1.12	0.85
	Standardized incidence (OECD)†	Total	1.10	1.19	1.84	1.53	0.72

*The overall standardized number per 1,000,000 population was calculated using the standard population in the year 2012 from the Korean Statistical Information Service in the Korea National Statistical Office.

^†^The overall standardized number per 1,000,000 population was calculated using the standard population in the year 2010 from the Organization for Economic Co-operation and Development (OECD) Statistics.

**Fig 2:**
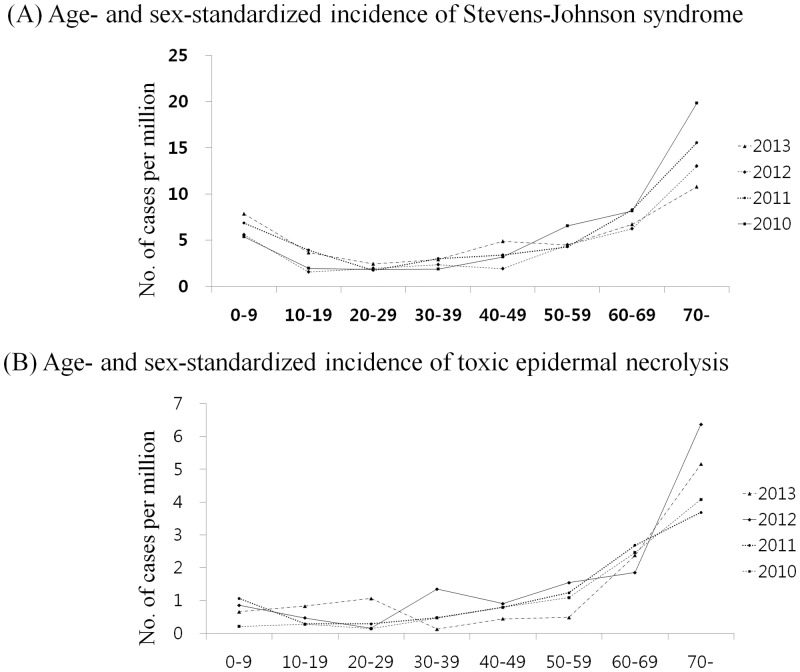
Standardized incidence rates and the percentage of death cases of SJS and TEN by age groups. The shape of incidence curves of Stevens-Johnson syndrome (A) and toxic epidermal necrolysis (B) were similar.

The characteristics of the study population are summarized in Table 2. Although there was year-to-year fluctuation, female cases were predominant in SJS while there was no significant sex difference in the numbers of the cases with TEN. There were significantly more cases aged 40 and older than those less than 40 years old in the occurrence both in SJS and TEN. However, the diseases occurred in people of any age group. More patients were managed in tertiary hospitals than in primary or secondary care facilities. However, statistical significance of the difference in the types of hospital diminished as time went on (2012 and 2013 for SJS and 2013 for TEN, respectively). The graph of age- and sex-standardized incidence rates of SJS and TEN by 10-year age group showed a similar right upward curve (Fig 2A and 2B). Group 20–29 years showed the lowest annual incidence of SJS (1.75–2.45 per million) and group 70 and older showed the highest (10.77–19.84 per million). As was similar with TEN that the annual incidence of group 20–29 years was 0.14–1.07 per million and that of group 70 and older was 3.68–6.37 per million.

**Table 2:** Characteristics of patients with Stevens-Johnson syndrome and toxic epidermal necrolysis.

Disease	Variable	Category	2010	2011	2012	2013	p for trend*
			No (%)	No (%)	No (%)	No (%)	
SJS	Total		235 (100.0)	246 (100.0)	200 (100.0)	257 (100.0)	0.88
	Sex (n, %)	Male	95 (40.4)	113 (45.9)	114 (57.0)	113 (44.0)	0.27
		Female	140 (59.6)	133 (54.1)	86 (43.0)	144 (56.0)	0.05
	Age group	0–39	69	96	68	97	0.08
		40-	166	150	132	160	0.16
	Types of hospitals (n, %)	Tertiary	150 (63.8)	161 (65.4)	104 (52.0)	130 (50.6)	
		Others	85 (36.2)	85 (34.6)	96 (48.0)	127 (49.4)	
	Health insurance (n, %)	NHI	221 (94.0)	228 (92.7)	192 (96.0)	240 (93.4)	
		MAS	14 (6.0)	18 (7.3)	8 (4.0)	17 (6.6)	
TEN	Total		45 (100.0)	53 (100.0)	73 (100.0)	58 (100.0)	0.09
	Sex (n, %)	Male	16 (35.6)	31 (58.5)	41 (56.2)	30 (51.7)	0.04
		Female	29 (64.4)	22 (41.5)	32 (43.8)	28 (48.2)	0.13
	Age group	0–39	8	13	19	16	0.05
		40-	37	40	54	42	0.55
	Types of hospitals (n, %)	Tertiary	32 (71.1)	35 (66.0)	50 (68.5)	33 (56.9)	
		Others	13 (28.9)	18 (34.0)	23 (31.5)	25 (43.1)	
	Health insurance (n, %)	NHI	41 (91.1)	51 (96.2)	67 (91.8)	54 (93.1)	
		MAS	4 (8.9)	2 (3.8)	6 (8.2)	4 (6.9)	

Abbreviation: SJS, Stevens-Johnson syndrome; TEN, toxic epidermal necrolysis; NHI, National Health Insurance; MAS, Medical Aid Service

*The test of trend (p for trend) for the pattern of total patients was conducted based on the annual Korean population size acquired from the Korea National Statistics office while the rest of categories were based on annual total number of cases.

The newly diagnosed SJS and TEN in the 2011 cohort was used to assess the percentage of death cases, related complications and direct medical costs. The characteristics of the retrospective cohort of patients diagnosed as SJS and TEN in 2011 are summarized in Table 3. The percentage of death cases during admission for SJS and TEN were 5.7% and 15.1%, respectively. Age-standardized mortality of SJS and TEN were 0.29 and 0.16 per million, respectively. Among the complications related with SJS or TEN, ocular sequelae was the most common (43.1% and 43.4% of SJS and TEN patients, respectively) followed by urethral sequelae (5.7% and 9.4% of SJS and TEN patients, respectively). There were only a few patients who sought medical care for other sequelae such as discoloration of the skin and nail problems. Total claim costs spent in 2011 on each episode were 2,915±4,843 USD and 9,726±16,873 USD for SJS and TEN, respectively. For SJS, costs reimbursed from the NHIS were 2,327±4,028 USD, and OOP expenses were 587±945 USD. For TEN, reimbursed costs were 7,934±14,360 USD, and OOP expenses were 1,709±2,884 USD. Interquartile ranges of the total claim costs for SJS and TEN were from 878 to 3,265 USD and from 1,793 to 8,872 USD, respectively. However, up to 56,147 and 77,504 USD were spent in treating SJS and TEN, respectively.

**Table 3:** Characteristics of the patients who had Stevens-Johnson syndrome or toxic epidermal necrolysis in 2011.

	SJS	TEN
Total (n)	246	53
Mortality (n, %)	14 (5.7)	8 (15.1)
Sequelae (n, %)		
Eye	106 (43.1)	23 (43.4)
Urethra	14 (5.7)	5 (9.4)
Others	3 (1.2)	1 (1.9)
Cost (mean, SD)*		
Total cost	2,915 (4,843)	9,726 (16,873)
Copayment	587 (945)	1,709 (2,884)
Reimbursed cost	2,327 (4,028)	7,934 (14,360)

*Costs were expressed in U.S. Dollars. The exchange rate between U.S. Dollars and Korean Won was 1:1,107.9 in 2011.

Abbreviation; SD, standard deviation

Finally, variables related to fatal outcomes were analyzed (Table 3). Sex and type of hospital were not different between the survivors and the deceased. The percentage of cases died of SJS and TEN significantly increased with age, particularly, in patients older than 40 years (Fig 2C and Table 4). Medical Aid Service recipients were more likely to die of TEN than National Health Insurance beneficiaries (11.8% for National Health Insurance beneficiaries vs. 100% for Medical Aid Service recipients, p = 0.02).

**Table 4:** Characteristics of the mortality cases in 2011 from Stevens-Johnson syndrome and toxic epidermal necrolysis.

	SJS (n = 14)		TEN (n = 8)	
	N (%)	p	N (%)	p
Sex				
Male	5/113 (4.4)	0.43	5/31 (16.1)	1.00
Female	9/133 (6.8)		3/22 (13.6)	
Age group				
0–19	0/59 (0)	<0.001*	0/7 (0)	<0.001*
20–39	0/37 (0)		1/6 (16.7)	
40–59	3/61 (4.9)		2/16 (12.5)	
60-	11/89 (12.4)		5/24 (20.8)	
Types of hospitals				
Tertiary	11/161 (6.8)	0.29	4/35 (11.4)	0.30
Others	3/85 (3.5)		4/18 (22.2)	
Health insurance				
National Health Insurance	13/228 (5.7)	0.98	6/51 (11.8)	0.02
Medical Aid Service	1/18 (5.6)		2/2 (100)	

*p-for trend

## Discussion

Medications are the most important risk factors for the development of SJS and TEN. Although carbamazepine, phenobarbital, phenytoin, and allopurinol were well-known causative agents of SJS and TEN [15], the number of newly marketed drugs could influence the incidence of these diseases [16]. Besides medications, changes in prescription patterns and insurance policies due to the notoriety of specific drugs could influence the incidence of serious adverse drug reactions [17]. However, it is difficult to survey the real world incidence of SJS and TEN because of their rarity. Several investigators have assessed the incidence of SJS and TEN recently, however, those estimates were mostly based on data from a single center and not on the general population [7–9]. In this study, we estimated the nationwide incidence of SJS and TEN using the National Health Insurance database in Korea which contained information about health care utilization of the entire national population. Therefore, this study provides more precise estimates of the population-based incidence of SJS and TEN. The age- and sex-standardized annual incidences estimated in this study were 3.96 to 5.03 in SJS and 0.94 to 1.45 in TEN per million and there was no significant change in annual incidence throughout the study periods. When comparing large scale epidemiologic studies showing the annual incidence of SJS and TEN as 1.2–6.0 and 0.4–1.2 per million, respectively [1–4], there were no remarkable increases in the incidence of SJS and TEN over the recent decades.

The mortality of SJS and TEN have been variously reported as 1–13 and 30–50%, respectively, and fatalities have been reported to occur in considerable numbers even after discharge from hospitals [2, 18–20]. In the current study, the percentage of death cases of SJS and TEN were 5.7% and 15.1%, respectively. While the mortality of SJS did not change much from the previous report, that of TEN was remarkably lower than the previous report although there were no definitive effective treatments introduced during the past two decades. Earlier diagnosis due to more information as well as improvement in intensive and supportive care could have contributed to this reduction in mortality of TEN.

The influence of age on the mortality of SJS and TEN was obvious (Table 4). Age is a well-known risk factor of mortality in SJS and TEN reflected by the Score of TEN (SCORTEN) [21]. As was expected, mortality of SJS and TEN in cases aged 40 and older was significantly higher than that of those less than 40 years in this study. Type of health insurance was related to the fatal outcome in TEN. However, the number of cases was too small to draw a conclusion.

If victims narrowly escape death, SJS and TEN can result in severe sequelae. Ocular disorders were the most common sequelae which required prolonged medical care. In the previous studies [22–24], the frequency of long-term ocular sequelae was about 20~50%. In the current study, 43.1% and 43.4% of the patients with SJS and TEN sought medical care due to ocular complications followed by urethral sequelae, the second most common complication occurring at a frequency of 5.7% and 9.4%, respectively. The frequency of urethral sequelae was higher in the TEN patients in agreement with the earlier reports [25]. Therefore, special attention should be given to urethral sequelae while managing patients with TEN. There were also a few cases with skin or nail problems, but there is a possibility of underestimation because only cases which needed regular medical care were included in this study.

**Fig 3:**
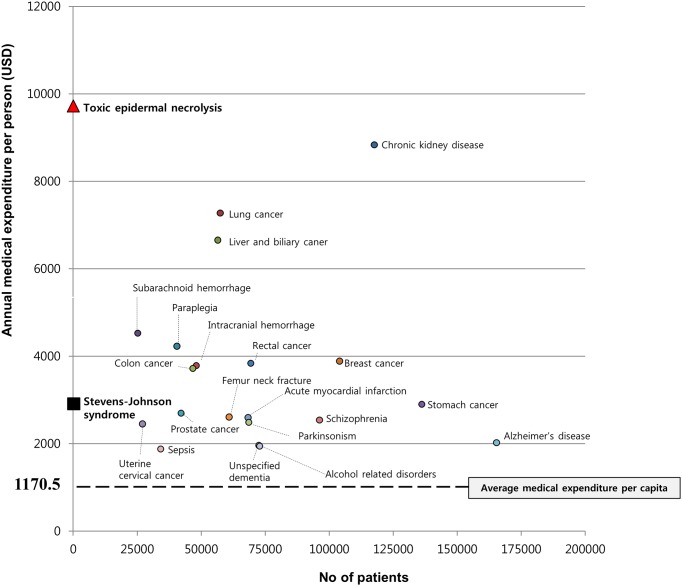
Comparison of annual medical expenditure per person between the top 20 reimbursed diseases and Stevens-Johnson syndrome and toxic epidermal necrolysis in 2011 in Korea. Although the numbers of patients were far smaller than that of the other diseases, the annual medical expenditure per person of SJS and TEN was comparable with that of the top 20 reimbursed diseases.

Medical costs can vary according to insurance systems and economic conditions of each country. Considering that the total medical expenditure per capita is about $1,700 in Korea, the total claims cost of managing SJS and TEN at $2,915 and $9,726 USD per person is substantial. When compared with the costs for managing top ten major diseases with high burden in Korea, it is clear that the individual burden of SJS and TEN are comparable to those diseases (Fig 3). Especially, the total claims cost spent in managing TEN was higher than the annual cost for the management of chronic kidney disease which is one of the most financially expensive chronic medical conditions in Korea. Moreover, the HIRA database does not include the costs for non-benefit items. The direct cost in the real world would be substantially higher when considering the costs for non-benefit items in the country such as intravenous immunoglobulin G and separate single room occupancy in treating SJS and TEN.

There are several limitations in the present study. First, the criteria for selecting cases were based solely on the International Classification of Diseases (ICD) codes without reviewing the medical records of the cases as we could not access the medical record of the patients. Thus, assessment of the cases by an expert panel was not possible. At the same time, SJS-TEN overlap syndrome could not be separated by ICD-10. However, by using ICD-10 which has separate diagnostic codes for SJS and TEN, it reduces the risk of misclassification [26]. Second, we could not identify the causative agent for each case. Third, there are some obstacles in generalizing the results of the present study because the incidence of adverse drug reactions like SJS and TEN is affected by many regional factors such as approval of a specific drug by the regional drug administration, prescription patterns of doctors, ethnic differences in susceptibility to certain diseases or adverse drug reactions, and the availability of medical services. Fourth, because the estimation of sequelae for SJS and TEN was based on the ICD codes inputted to the database as a result of hospital visits, more frequent but clinically less severe sequelae would be missed in the analysis. Fifth, this study failed to take into account various other confounding factors, such as underlying diseases, co-administered medications, and socioeconomic status, which could potentially affect the courses of SJS and TEN. Sixth, variables related to the management such as receiving intensive care or specific treatment regimen like intravenous immunoglobulin could not be investigated because we could not access the data. Seventh, as we included the cases who utilized healthcare services for SJS and TEN, there was possibility that part of mild cases who did not received healthcare service would be excluded. Lastly, the percentage of death cases could be underestimated because this study reflected the deceased cases in the hospital only. In fact, a recent large population-based follow-up study reported remarkable numbers of deaths after discharge [20]. Despite these limitations, the HIRA Service database in Korea, which includes data on the healthcare of all people residing in Korea, could be very helpful in estimating the incidence of rare but severe diseases such as SJS and TEN.

## Conclusions

We estimated the most recent incidence of SJS and TEN from the entire national administrative database in Korea. Our data suggest that SJS and TEN are infrequent but constantly arise throughout the years whereas the mortality of TEN seems to have decreased. Ocular and urethral sequelae were the most frequent long-term sequelae of SJS and TEN which required additional medical care after discharge.
